# Clinical significance of *TP53, BIRC3, ATM* and *MAPK-ERK* genes in chronic lymphocytic leukaemia: data from the randomised UK LRF CLL4 trial

**DOI:** 10.1038/s41375-020-0723-2

**Published:** 2020-02-03

**Authors:** Stuart J. Blakemore, Ruth Clifford, Helen Parker, Pavlos Antoniou, Ewa Stec-Dziedzic, Marta Larrayoz, Zadie Davis, Latha Kadalyayil, Andrew Colins, Pauline Robbe, Dimitris Vavoulis, Jade Forster, Louise Carr, Ricardo Morilla, Monica Else, Dean Bryant, Helen McCarthy, Renata J. Walewska, Andrew J. Steele, Jacqueline Chan, Graham Speight, Tanja Stankovic, Mark S. Cragg, Daniel Catovsky, David G. Oscier, Matthew J. J. Rose-Zerilli, Anna Schuh, Jonathan C. Strefford

**Affiliations:** 1 Academic Unit of Cancer Sciences, Faculty of Medicine, University of Southampton, Southampton, UK; 2 Department I of Internal Medicine, Centre of Excellence in Aging Research, University of Cologne, Cologne, Germany; 3 Oxford National Institute for Health Research Biomedical Research Centre and Department of Oncology, University of Oxford, Oxford, UK; 4 Department of Molecular Pathology, Royal Bournemouth Hospital, Bournemouth, UK; 5 Genetic Epidemiology and Bioinformatics, Faculty of Medicine, University of Southampton, Southampton, UK; 6 Division of Molecular Pathology, The Institute of Cancer Research, London, UK; 7 Oxford Gene Technology, Begbroke Science Park, Begbroke, Oxfordshire UK; 8 Institute of Cancer and Genomic Sciences, College of Medical and Dental Services, IBR West, University of Birmingham, Birmingham, UK; 9 Antibody & Vaccine Group, Centre for Cancer Immunology, Cancer Sciences Unit, Faculty of Medicine, University of Southampton, Southampton General Hospital, Southampton, UK

**Keywords:** Chronic lymphocytic leukaemia, Genetics research, Cancer genomics

## Abstract

Despite advances in chronic lymphocytic leukaemia (CLL) treatment, globally chemotherapy remains a central treatment modality, with chemotherapy trials representing an invaluable resource to explore disease-related/genetic features contributing to long-term outcomes. In 499 LRF CLL4 cases, a trial with >12 years follow-up, we employed targeted resequencing of 22 genes, identifying 623 mutations. After background mutation rate correction, 11/22 genes were recurrently mutated at frequencies between 3.6% (*NFKBIE*) and 24% (*SF3B1*). Mutations beyond Sanger resolution (<12% VAF) were observed in all genes, with *KRAS* mutations principally composed of these low VAF variants. Firstly, employing orthogonal approaches to confirm <12% VAF *TP53* mutations, we assessed the clinical impact of *TP53* clonal architecture. Whilst ≥ 12% VAF *TP53*mut cases were associated with reduced PFS and OS, we could not demonstrate a difference between <12% VAF *TP53* mutations and either wild type or ≥12% VAF *TP53*mut cases. Secondly, we identified biallelic *BIRC3* lesions (mutation and deletion) as an independent marker of inferior PFS and OS. Finally, we observed that mutated *MAPK-ERK* genes were independent markers of poor OS in multivariate survival analysis. In conclusion, our study supports using targeted resequencing of expanded gene panels to elucidate the prognostic impact of gene mutations.

## Introduction

The application of new technologies continues to reveal the biological basis for the clinical heterogeneity apparent within CLL [1–3]. In particular, next generation sequencing of large patient cohorts has led to the discovery of recurring genomic mutations that cluster into distinct biological signalling pathways. Mutations of specific genes including *TP53* [4–10]*, ATM* [9, 11–14]*, BIRC3* [9, 15, 16]*, SF3B1* [9, 17–20]*, NOTCH1* [1, 9, 15, 17, 20–23], *RPS15* [2, 24]*, EGR2* [25, 26] and *KRAS* [27, 28] are associated with poorer outcome, especially shorter time to first treatment or overall survival (OS). However, numerous factors influence the clinical significance of a driver mutation in an individual patient. These include clinical status, immunogenetic background, clone size, the presence of biallelic abnormalities and co-existing driver mutations or copy number alterations (CNAs). The clinical importance of these potentially confounding factors is most easily established in context of large clinical trials with long follow-up and where data on numerous biomarkers are available. One such study is the phase III UK LRF CLL4 trial (NCT 58585610) that randomly assigned 777 patients to fludarabine (FDR) or fludarabine plus cyclophosphamide (FC) for six courses, or chlorambucil (CHL) for 12 courses, with the primary endpoint of OS, and secondary endpoints of response rates, progression-free survival, toxic effects and quality of life [29]. The trial demonstrated superior response rates and progression-free survival (PFS) for FC-treated patients compared with those patients treated with FDR or CHL. Previous genomic analysis of this trial has shown *TP53* [8], *SF3B1* [17], *NOTCH1* (coding [17] and non-coding [21]), *ATM* plus del(11q) [12] and *EGR2* [26] lesions to have prognostic significance in multivariate analysis (MVA) and of *RPS15* [24] in univariate analysis. The importance of data from CLL4 may be questioned given the studies showing the superior efficacy of FC plus an anti-CD20 antibody (FCR) compared with chemotherapy alone, with the exception of patients with a *NOTCH1* mutation [20], and emerging data suggesting the superiority of novel agents compared with chemotherapy-based regimens. However, the observation that *TP53*, *SF3B1* and *RPS15* mutations remain poor risk factors in the German CLL8 trial comparing FCR vs. FC [20] and the continuing global need for chemotherapy in CLL for the foreseeable future, indicate that genomic data from the UK CLL4 trial will continue to have clinical relevance.

Accordingly, we performed targeted resequencing on all available pre-treatment samples (*n* = 499) from the CLL4 trial to investigate the incidence, clinico-biological associations and prognostic impact of a panel of 22 genes recurrently mutated in CLL (study overview in Fig. S1). Important findings include the failure of <12% VAF *TP53* mutations (1.97–11.18% variant allele frequency [VAF]) to influence PFS or OS, the importance of 11q deletions on PFS and OS in the context of *ATM* and *BIRC3* mutations, and the reduced OS associated with mutations in the *MAPK-ERK* genes: *BRAF, KRAS* and *NRAS*.

## Methods

### Patients and molecular assays

We studied 499 patient samples taken at randomisation [29]. Patients were diagnosed using the iwCLL guidelines [30], with informed consent obtained in accordance with the declaration of Helsinki. This study was approved by national/regional research ethics committees. The average lymphocyte percentage of the total white cell count in pre-treatment blood samples was 83.8%. To confirm high tumour load, CD19/CD5 positivity from cases with available flow-cytometry data were compared with their matched average lymphocyte percentage (*n* = 233), with an agreement bias of −0.8% (Fig. S2). Our study cohort did not significantly differ from the entire trial cohort in terms of: treatment allocation, CNAs, age, gender, disease stage, ZAP70/CD38 expression, or IGHV status (Table S1). The assessment of established biomarkers was performed as described [31]. All published genetic and biological data on CLL4 patients for genes: *TP53* [8]*, ATM* [12, 13]*, BIRC3* [12]*, NOTCH1* [17] (+3′UTR [21]) and *SF3B1* [17], and CNAs: 13q deletion, 17p deletion, 11q deletion and trisomy 12 (5%, 10%, 5% and 3% clone size cut-offs, respectively [31]) were integrated into this study, as well as telomere length [32] and levels of prolymphocytes [33].

### Targeted resequencing, bioinformatics analysis, variant filtering and validation

Mutations in 22 genes were analysed in all 499 patients (TruSeq Custom Amplicon, Illumina, San Diego, CA, USA) (Table S2). Libraries were generated from 250 or 50 ng (dependent on the amount of available starting material) of DNA according to manufacturer’s instructions. The average sequencing yield after Illumina processing (MiSeq, paired-end, 2 × 150 bp) from 28 runs was 6.9 Gbp, with a mean read depth of >1000 × (range 502–7948) across all targeted genes, with only nine amplicons below a mean read depth of 1000 (range 502–987) (Fig. S3).

At this depth subclonal mutations can be detected at the 2% level, assuming a minimum observation of four sequencing reads containing the variant base, a Q50 phred like base quality score (*p*(detected) = 99.999) and a cumulative binomial distribution for n read depth $[\frac{{N!}}{{n!\left( {N - n} \right)!}}p^n(1 - p)^{N - n}]$. In addition, six variants below 2% were included, since the number of sequencing reads in the variant base were more than ten times the assumed minimum observation (range 50–126), and the total read depth exceeded 2000 reads in all cases (range 2582–6389). Bioinformatic data processing of variants was conducted as previously described [14].

All mutations included in this study are listed in Table S3. As the CLL4 cohort lacked germ-line DNA, we only considered variants previously observed as somatically acquired in CLL [1, 2, 14] or annotated in COSMIC (v70) [34], except for specific circumstances regarding *TP53, ATM, BIRC3* and *NOTCH1*. For *TP53*, additional mutations annotated in IARC were re-introduced [35]. Pathogenic *ATM* variants were included if; they were observed in AT families as pathogenic (LOVD [https://databases.lovd.nl/shared/genes/ATM]), they were evolutionary rare missense [36], or were somatically acquired in CLL [13] (Table S4). However, this variant strategy does not fully preclude *ATM* variants that exist in germ-line material. For *BIRC3*, only truncating mutations were included [9]. *NOTCH1* PEST domain mutations not predicted to result in protein truncation were removed. All candidate variants were visually inspected in Integrated Genomics Viewer [37]. Genes were defined as recurrent using Tumour Portal (www.tumorportal.org/power), with the background mutation rate for CLL stated on the website, and the number of cases in the study (*n* = 499) inputted. Mutations were stratified using Sanger sequencing threshold of 12% [5, 9].

Thirty-one percent (194/623) of mutations were validated using orthogonal approaches, including Sanger (*n* = 120) and Ion Torrent (19 low-level *TP53* mutations) sequencing, hybridization-based gene enrichment with subsequent sequencing (*n* = 27) and ddPCR (*SF3B1* p.K700E [n = 11], *NOTCH1* p.P2415fs [*n* = 19]). One hundred percent of variants were confirmed using this approach. An excellent agreement between TruSeq and orthogonal-derived VAFs was also observed, with an agreement bias of 0.02% (Fig. S4).

### Statistical analysis

Fisher’s exact tests were performed for co-occurrence analysis between mutated genes and clinical features. PFS and OS were assessed from randomisation using Kaplan Meier (KM) and Log rank analysis. PFS was defined as time from randomisation to progression (i.e. relapse needing further treatment) or death, or to last follow-up date (Oct 2010; final CLL4 PFS update). OS was defined as time from randomisation to death or to last follow-up date for survivors (August 2016, final CLL4 OS update). Multivariate Cox Proportional Hazard models were generated for OS and PFS using backwards selection (*P* < 0.05), to test the confounding effect of multiple prognostic variables. The Bland–Altman test was used to test agreement between multiple factors, reporting the agreement bias, which is the mean difference between two measurements. All reported *P* values were two-sided and results were considered significant at the 5% level, using multiple hypothesis testing when appropriate (Benjamini and Hochberg method [38]). Statistical analysis was conducted using R v3.3.0, SPSS v23 (IBM), and Prism v6.0 g (GraphPad).

## Results

### Distribution of somatic mutations

**Fig. 1:**
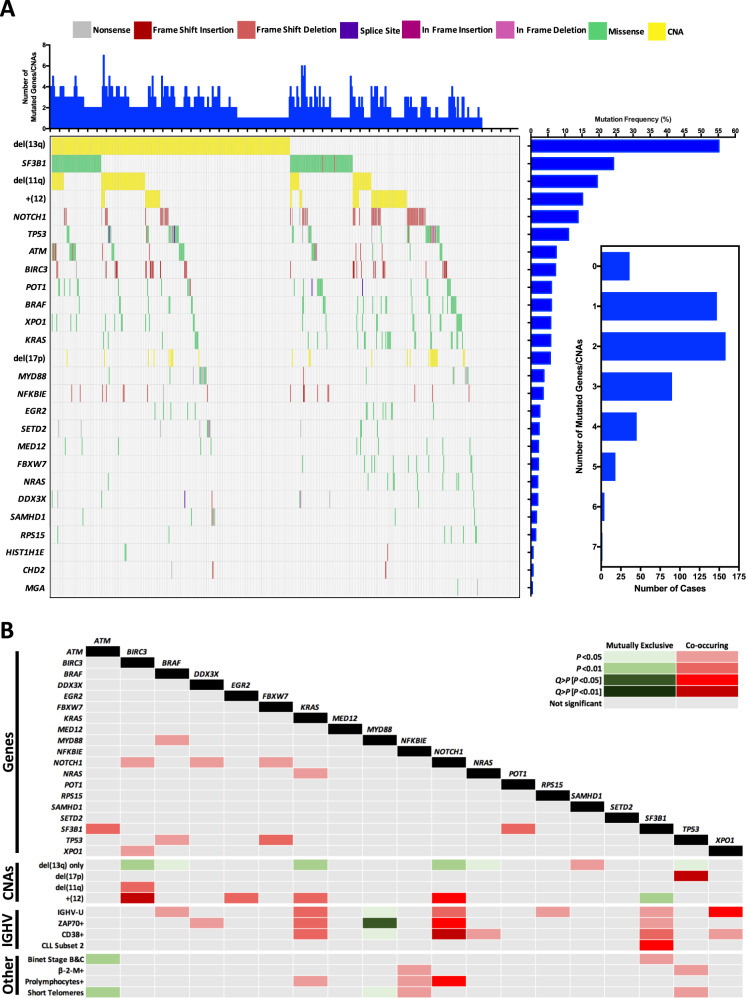
Mutation landscape and co-occurrence associations of the CLL4 cohort. **a** Mutational landscape of CLL4. In the Waterfall plot, known recurrently mutated genes and copy number alterations are shown, hierarchically clustered by mutation frequency (vertical bar chart, right). The mutation burden captured by the study is shown in the bar chart above the heat map. Mutation types are depicted in the above key. The inset vertical bar chart represents the distribution of the number of mutated genes/CNAs per case. **b** Co-occurrence of all available clinico-biological features from the CLL4 clinical trial. The co-occurrence (red) or mutual exclusivity (green) is plotted per interaction in the graph based on the level of significance (from light to dark: *P* < 0.05, *P* < 0.01, *Q* > *P* [*P* < 0.05], *Q* > *P* [*P* < 0.01]).

We identified 623 mutations (mean = 1.25, min/max = 0/7 per patient) in 335 patients, 398 ≥ 12% VAF and 225 < 12% VAF, with 93% of the entire cohort harbouring ≥1 mutation or CNA (Fig. 1a). Ninety-seven patients without any established CNAs carried 124 mutations (mean = 1.28, min/max = 0/6 per patient), with 22 patients lacking any mutations or CNAs. After background correction (0.5/Mb, ≥3% recurrence, Table S5), 11/22 genes were recurrently mutated at frequencies between 3.6% (*NFKBIE*) and 24% (*SF3B1*), (Fig. 1a, Table S3, Fig. S5). 121 samples harboured 134 *SF3B1* mutations; 46.3% were the p.K700E variant and 30.6% were other hotspot variants (p.K666X, p.H662X, pG740E, p.G742D). Two or more *SF3B1* mutations were identified in 12 patients (Fig. S6), with six cases harbouring multiple *SF3B1* mutations present with different VAFs, suggesting the presence of multiple mutated sub-clones. 69 *NOTCH1* mutated patients were identified (13.8%), with 61 mutations in exon 34 (50/61 p.2514 fs) and 9 in the 3′UTR. Fifty-five patients carried 59 IARC-annotated *TP53* mutations (exons 4–11, 88% in exons 5–8). Forty pathogenic *ATM* mutations were observed in 37 cases, without evidence of any mutational hotspots. *BIRC3*, *POT, BRAF, XPO1* and *KRAS* were mutated in 7.2, 6, 6, 5.8 and 5.8% of cases, respectively. Thirty-eight cases harboured a mutation in *BRAF*, with 7 (18.4%) having the p.V600E variant (Fig. S7).

### Clinico-biological features of recurrently mutated genes

Next, we determined statistical associations between these gene mutations, and expansive clinico-biological features, using the Fisher’s Exact test (*n* = 1293 tests, Fig. 1b). One hundred twenty-six associations were observed, including 15 high- (FDR, *Q* > *P* [*P* < 0.05]), 35 medium- (*P* < 0.01) and 76 low-confidence associations (*P* < 0.05). Significant associations between mutations were found in only 10/171 possible associations, such as *NOTCH1* + 3′UTR with *BIRC3* (*P* = 0.02) and *FBXW7* (*P* = 0.01), as well as *BRAF* with *TP53* (*P* = 0.03) (Fig. 1b, Table S6).

### Distribution of ≥12% VAF and <12% VAF mutations

**Fig. 2:**
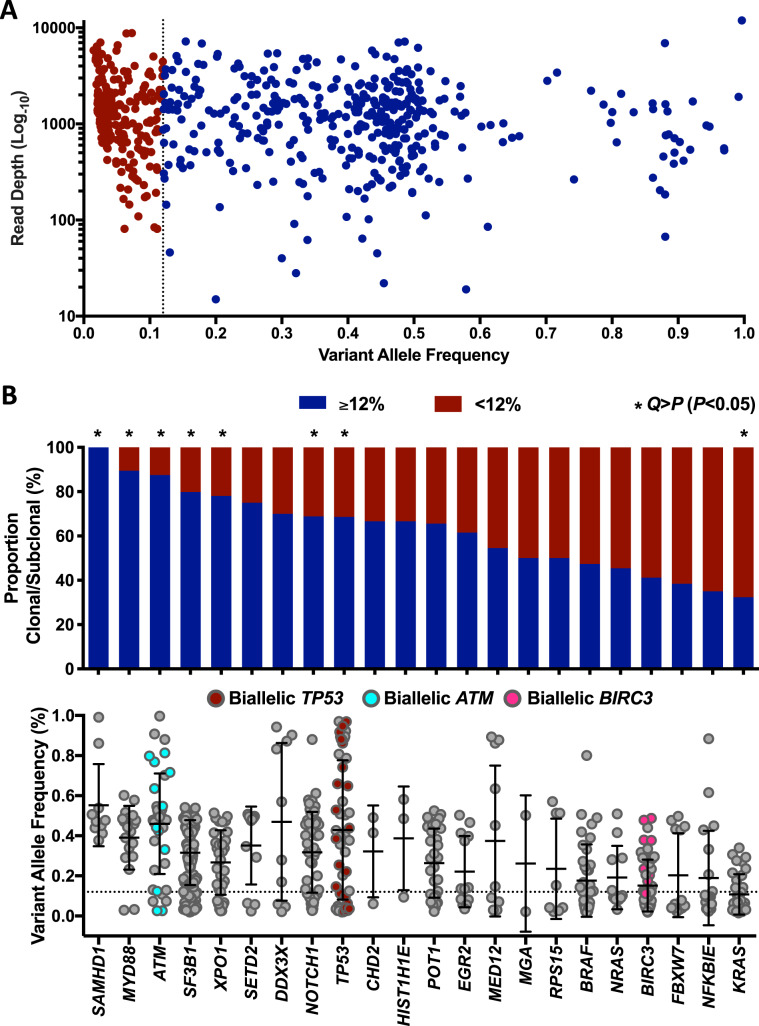
CLL4 mutation architecture. **a** Distribution of mutation variant allele frequency. Scatter plot of all variants by read depth and VAF (red dots = < 12% VAF [left of dotted line], blue dots = > 12% VAF). **b** Distribution of ≥12% and <12% variants. Top: Proportion of ≥12% and <12% variants ranked by highest proportion of ≥12% VAF variants. Two-way binomial distribution used to test whether genes contained significantly more ≥12% VAF or <12% VAF mutations, with asterisks representing genes which retained significance after multiple hypothesis testing (*Q* > *P [P* < 0.05]). Bottom: VAF distribution of variants per gene. Variants with loss of the other allele (identified by FISH), shown in red for biallelic *TP53*, turquoise for biallelic *ATM* and pink for biallelic *BIRC3.*

Next, we classified mutations as Sanger positive (≥12% VAF) or negative (<12% VAF) by accounting for the impact of tumour purity on VAF. Initially, we studied 233 patients with tumour purity derived from CD5/CD19 flow cytometry. Raw VAFs were compared with purity-adjusted VAFs across all variants (*n* = 288), including <12% VAFs (*n* = 98), and showed an agreement bias of only 5% (Fig. S8A), which was even lower for <12% VAF mutations (agreement bias < 0.82%, Fig. S8B). Therefore, we analysed all raw VAFs, and observed three variant populations: those found at <12% VAF (1.49–11.56%, *n* = 225), those at larger subclonal or clonal levels (12.06–58.15%, *n* = 356), and those concomitant with deletion events (60.19–99.66%, *n* = 42) (Fig. 2a). *SAMHD1* mutations were exclusively composed of ≥12% VAF (55.3% mean VAF), while *ATM, MYD88, NOTCH1, SF3B1, TP53* and *XPO1* mutations were found to be contain a significant majority of ≥12% VAF mutations. *KRAS* mutations were more likely to be composed of low VAF variants, with a mean VAF of 10.7% (two-way binomial test, False Discovery Rate [FDR], *Q* > *P* [*P* < 0.05]) (Fig. 2b).

### Univariate impact of mutated genes on PFS and OS

**Fig. 3:**
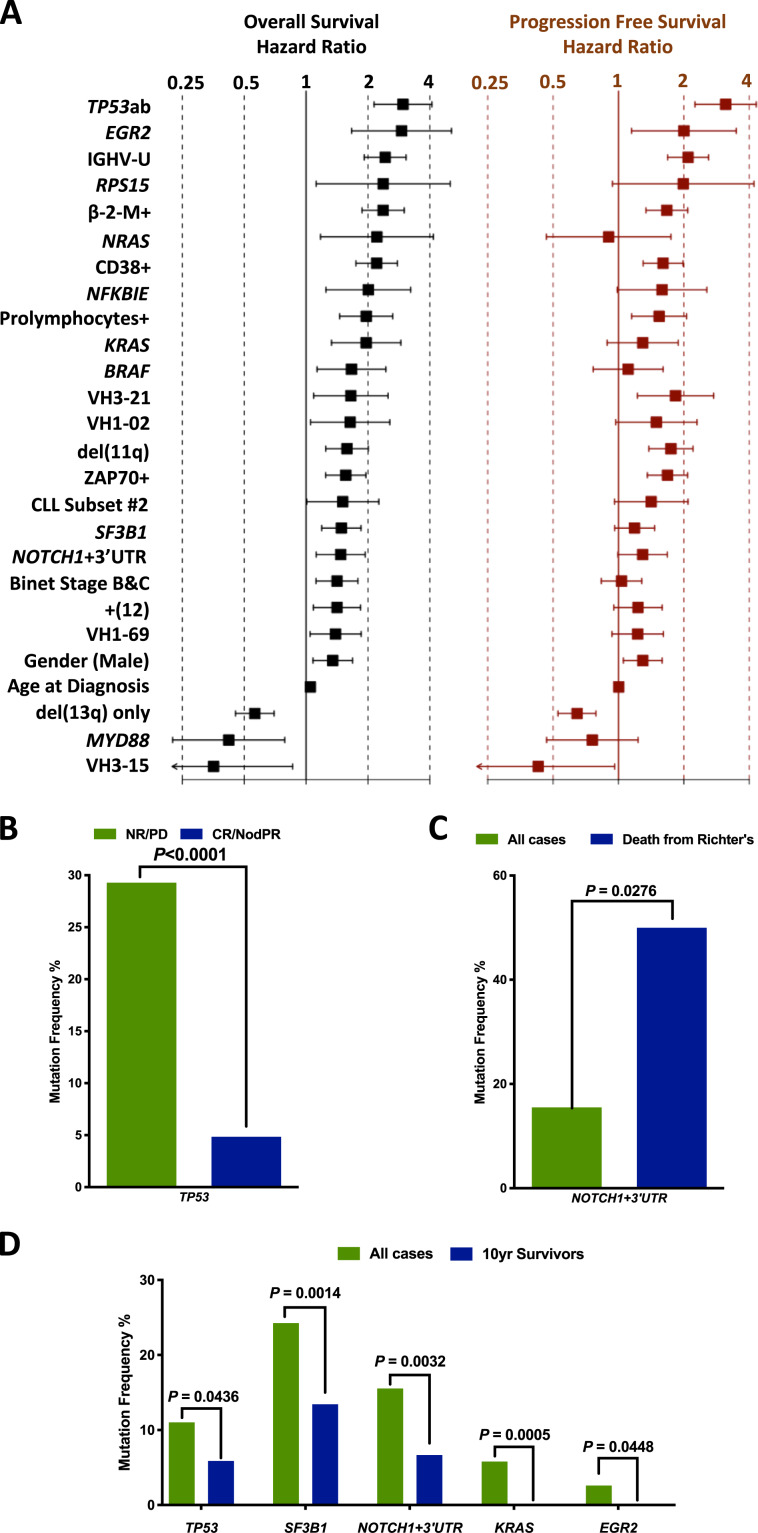
Clinical outcome of mutated genes, CNAs and clinical features in CLL4. **a** Forest plot showing the hazard ratios of 26 significant variables for either overall survival (left; black) or progression-free survival (right; red) in univariate survival analysis. Variables sorted by the hazard ratio values for overall survival. **b** Bar chart showing the mutation frequency difference between *TP53*mut cases who achieved CR/NodPR or NR/PD. **c** Bar chart showing the *NOTCH1* + 3′UTR mutation frequency in relation to Death from Richter’s syndrome. **d** Bar chart showing the mutation frequency in relation to patients termed ‘long-term survivors’ for *TP53, SF3B1, NOTCH* +3′UTR, *KRAS* and *EGR2.*

Clinico-biological features and gene mutations associated with PFS and OS in univariate Cox Proportional Hazards analysis are shown in Fig. 3a. Gene mutations in *TP53* (with/without del(17p), termed ‘*TP53*ab’) and *EGR2* were associated with reduced PFS (Fig. 3a, Table 1 and Fig. S9). *TP53*ab, and recurrent mutations in *SF3B1, NOTCH1* (+3′UTR)*, EGR2, RPS15, NFKBIE, BRAF, KRAS* and *NRAS* were associated with reduced OS (Fig. 3a, Table 1 and Fig. S10). As expected, mutations in *MYD88* were confined to IGHV-mutated (IGHV-M) cases, having no significant impact on OS in this subgroup of patients (Fig. S11). In addition, *TP53* mutations were associated with poor response (Fig. 3b), *NOTCH1* + 3′UTR mutations were associated with death from Richter’s syndrome (Fig. 3c), whilst *TP53, SF3B1, NOTCH1* + 3′UTR, *KRAS* and *EGR2* were significantly associated with < 10 year survival (Fig. 3d). Other significant associations are included in Figs. S12 and S13.

**Table 1:** Univariate survival association analysis for overall survival and progression-free survival in CLL4.

Variable	Status	Overall survival									Progression-free survival								
		Total	Events	Median (years)	LCI	UCI	HR	LCI	UCI	*P* Value	Total	Events	Median (years)	LCI	UCI	HR	LCI	UCI	*P* Value
*BRAF*	Unmutated	469	383	6	5.39	6.55	–	–	–	–	469	429	2	1.83	2.33	–	–	–	–
	Mutated	30	28	2.87	6.25	7.5	1.66	1.13	2.44	0.009	30	30	2.12	1.42	3.33	1.11	0.77	1.61	0.586
*EGR2*	Unmutated	486	398	5.95	5.08	6.39	–	–	–	–	486	446	2.08	22	28	–	–	–	–
	Mutated	13	13	3.45	3.85	–	2.91	1.67	5.1	<0.0001	13	13	0.415	10	NA	2	1.15	3.49	0.012
*KRAS*	Unmutated	470	383	5.89	5.32	6.44	–	–	–	–	470	430	2.04	22	28	–	–	–	–
	Mutated	29	28	3.83	2.79	6.86	1.96	1.33	2.89	<0.001	29	29	1.92	18	35	1.29	0.89	1.89	0.18
*MYD88*	Unmutated	480	401	5.67	4.96	6.25	–	–	–	–	480	442	1.96	21	27	–	–	–	–
	Mutated	19	10	10.3	7.15	–	0.42	0.22	0.79	0.005	19	17	2.67	27	68	0.76	0.47	1.23	0.262
*NFKBIE*	Unmutated	481	393	5.9	5.32	6.47	–	–	–	–	481	441	2	22	28	–	–	–	–
	Mutated	18	18	3.61	2.77	6.98	2.01	1.25	3.23	0.003	18	18	1.83	16	35	1.59	0.99	2.55	0.054
*NOTCH1* **+** 3′UTR	Unmutated	375	306	6.22	5.56	6.73	–	–	–	–	430	391	2.17	22	28	–	–	–	–
	Mutated	69	62	4.28	3.62	6.03	1.47	1.12	1.94	0.005	69	68	1.92	17	30	1.24	0.96	1.61	0.099
*NRAS*	Unmutated	489	401	5.88	5.23	6.44	–	–	–	–	489	450	2	22	28	–	–	–	–
	Mutated	10	10	4.24	2.05	–	2.21	1.18	4.16	0.011	10	9	2.54	19	NA	0.9	0.47	1.75	0.758
*RPS15*	Unmutated	492	404	5.86	5.3	6.42	–	–	–	–	492	452	2.08	22	28	–	–	–	–
	Mutated	7	7	2.89	2.18	–	2.37	1.12	5.03	0.02	7	7	1.75	3	NA	1.99	0.94	4.21	0.067
*SF3B1*	Unmutated	378	301	6.33	5.64	6.99	–	–	–	–	378	344	2.17	22	29	–	–	–	–
	Mutated	121	110	4.49	3.92	5.65	1.48	1.19	1.85	<0.001	121	115	1.92	17	28	1.19	0.96	1.47	0.112
*TP53*	Unmutated	444	360	6.15	5.64	6.7	–	–	–	–	451	413	2.17	23	29	–	–	–	–
	Mutated	55	51	2.65	1.47	3.87	2	1.49	2.69	<0.001	48	46	0.5	5	15	1.95	1.27	2.34	<0.0001

### Clinical relevance of TP53 deletions and mutations

**Fig. 4:**
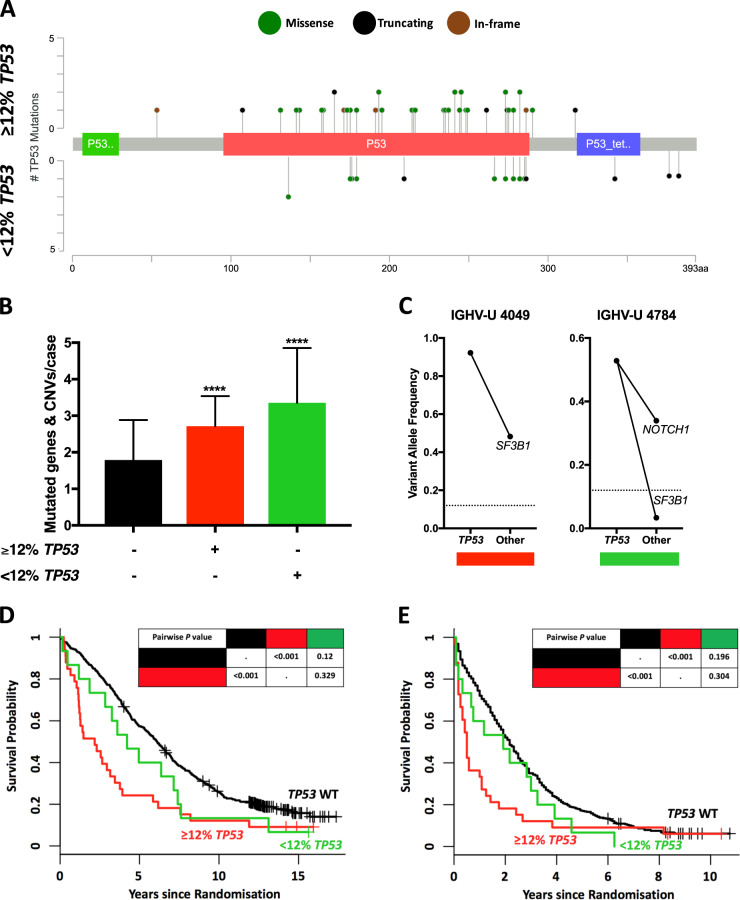
Clinical relevance of <12% VAF TP53 mutations in CLL4. **a** Mutation Lolliplot displaying the *TP53* mutations observed in CLL4, stratified by Sanger sequencing threshold. **b** Mutated genes/CNVs per *TP53*mut subgroup. One-way ANOVA conducted vs. *TP53*wt cases. **c** Examples of In-going and out-going edges drawn from each *TP53*mut subgroup, with patient ID number and IGHV status defined above each graph. **d** OS pairwise KM plot comparing ≥ 12% VAF *TP53*mut cases (red), <12% VAF *TP53*mut cases(green), and *TP53*wt cases (black). **e** PFS pairwise KM plot comparing ≥ 12% VAF *TP53*mut cases (red), <12% VAF *TP53*mut cases(green), and *TP53*wt cases (black). Inset table in D&E displays pairwise log rank *P* values between each variable vs. wild type.

*TP53* mutations below the threshold of Sanger sequencing have been associated with inferior survival in retrospective analysis of institutional cohorts [5, 9]. We observed 59 *TP53* mutations in 55 patients (Fig. 4a); all of those tested (*n* = 51) were confirmed using orthogonal approaches (Table S3). These <12% *TP53* mutated cases were enriched for *BRAF* and *FBXW7* mutations (Table S7). *TP53* mutations could be further subdivided into those with < 12% VAF (*n* = 16) or ≥12% VAF (*n* = 43), with no difference in the site or type of *TP53* mutation between subgroups (Fig. 4a). After including 17p FISH data, 58 *TP53*ab patients were identified, divided into cases with sole 17p deletions (*n* = 3), isolated *TP53* mutations (*n* = 27) or both (*n* = 23). Five *TP53* mutated cases lacked FISH data.

Next, we assessed the genomic complexity of *TP53*mut cases. Both <12% VAF and ≥12% VAF *TP53*mut groups had increased mutation/CNA frequency in comparison to *TP53*wt cases (both *P* < 0.001) (Fig. 4b). To further understand the complexity of these two patient subgroups, we inferred the evolutionary history of *TP53*mut cases as previously described in CLL [2]. Both <12% VAF and ≥12% VAF cases exhibited the same heterogeneous pattern of co-existing mutations, where *TP53* mutations were present at higher, or lower VAFS than concomitant driver mutations (Table S8, Figs. 4c and S14).

Lastly, we assessed the clinical impact of <12% VAF and ≥12% VAF *TP53*mut subgroups in pairwise KM analysis. ≥12% VAF *TP53*mut were associated with reduced PFS and OS compared with cases with wild-type *TP53* (≥12% *TP53*mut = OS: median = 2.18 years vs. 6.11 years, *P* < 0.001, PFS: median = 0.5 years vs. 2.17 years, *P* < 0.001). In contrast, we could not demonstrate a significant difference between the <12% VAF *TP53*mut cases and either the wild type or ≥12% VAF *TP53*mut patients for PFS or OS (<12% *TP53*mut = OS: median = 4.21 years vs. 6.11 years, *P* = 0.12, PFS: median = 1.92 years vs. 2.17 years, *P* = 0.196) (Fig. 4d, e). These observations held true in 17p deletion stratified analysis (Fig. S15), confirming the importance of *TP53*mut clone size on survival in this cohort. Stratified < 12% VAF vs. ≥ 12% VAF analysis for other genes with sufficient mutated cases in this cohort can be found in Figs. S16 and S17.

### Biallelic BIRC3 deleted patients infer reduced OS in comparison to sole 11q deleted patients

**Fig. 5:**
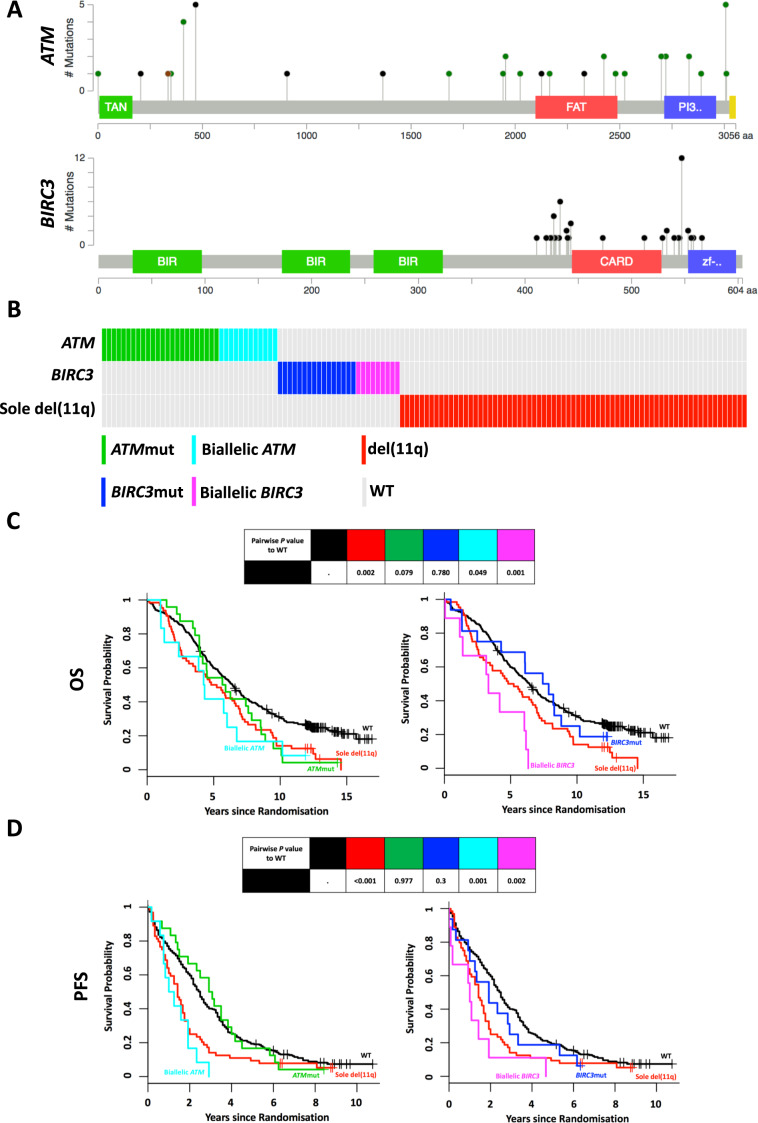
Importance of 11q deletion in the context of *ATM* and *BIRC3* mutations in CLL4. **a** Mutation Lolliplot of *ATM* (upper) and *BIRC3* (lower) mutations observed in CLL4. **b** Heat map of *ATM* and *BIRC3* mutated cases stratified by 11q deletion status. **c** OS pairwise KM plot comparing mutated *ATM* (left) and *BIRC3* (right) in the context of 11q deletion. **d** PFS pairwise KM plot comparing mutated *ATM* (left) and *BIRC3* (right) in the context of 11q deletion. Inset table in C&D displays pairwise log rank *P* values between each variable vs. wild type for combined pairwise KM analysis of *ATM* and *BIRC3* in the context of 11q deletion.

Although neither *ATM* nor *BIRC3* mutations, regardless of their VAF (Figs. S16 and S17), were associated with reduced PFS or OS in univariate survival analysis (Figs. S12 and S13), it has previously been demonstrated that the impact of these mutations may be dependent on the presence of a concomitant 11q deletion [12, 39]. Therefore, we performed an integrated analysis of the clinical impact of *ATM* and *BIRC3* mutations in the context of 11q deleted CLL. *ATM* Mutations spanned the entire gene, whilst those targeting *BIRC3* were restricted to the CARD domain, as previously shown [9, 11–13, 39] (Figs. 5a and  S7). Importantly, *ATM* and *BIRC3* mutations were mutually exclusive in our series (Fig. 5b), suggesting that these mutations may define subgroups of 11q deleted CLL. Deletions of 11q were identified using a FISH probe, which encompasses the *ATM* but not the *BIRC3* locus. Accordingly, concomitant *BIRC3* loss was defined from previously published SNP6.0 data [12], or where additional DNA was available (*n* = 21), using shallow WGS (positive cases presented in Fig. S18). Cases (*n* = 135) were then categorised into five distinct subgroups: sole 11q deletion (*n* = 71), biallelic *ATM* abnormalities (abs) (*n* = 12), biallelic *BIRC3* abs (*n* = 9), sole *ATM* mutations (*n* = 24) and sole *BIRC3* mutations (*n* = 19).

After omitting ten cases with co-existing *TP53*ab [12], we conducted pairwise KM analysis for these five groups compared with cases with no 11q abnormality. (Figs. 5c, d and S19). For both PFS and OS, sole 11q deletion (PFS: median = 1.4 years vs. 2.5 years, *P* < 0.0001, OS: median = 4.8 years vs. 6.4 years, *P* = 0.002), as well as biallelic *ATM* (PFS: median = 1 year vs. 2.5 years, *P* = 0.001, OS: median = 4.2 years vs. 6.4 years, *P* = 0.049) and biallelic *BIRC3* (PFS: median = 1 year vs. 2.5 years, *P* < 0.0001, OS: median = 3.3 years vs. 6.4 years, *P* = 0.001), were associated with a significantly reduced survival.

The outcome of cases with biallelic abs was then compared with those with del(11q) only. There were no significant differences in PFS (biallelic *ATM* vs. 11q = 1 year vs. 1.4 years, *P* *=* 0.336; biallelic *BIRC3*: 1 year vs. 1.4 years, *P* = 0.178); however cases with biallelic *BIRC3* abs had a significantly reduced OS, whilst cases with biallelic *ATM* abs did not significantly differ in median survival times compared with sole 11q deleted cases (biallelic *ATM* vs. 11q = 4.2 years vs. 4.8 years, *P* *=* 0.493; biallelic *BIRC3*: 3.3 years vs. 4.8 years, *P* = 0.03). This suggests that biallelic loss of *BIRC3* represents the subgroup of 11q deleted CLL with the worst outcome following initial treatment with chemotherapy.

### MAPK-ERK pathway members: BRAF, KRAS and NRAS, all infer poor OS in CLL4

**Fig. 6:**
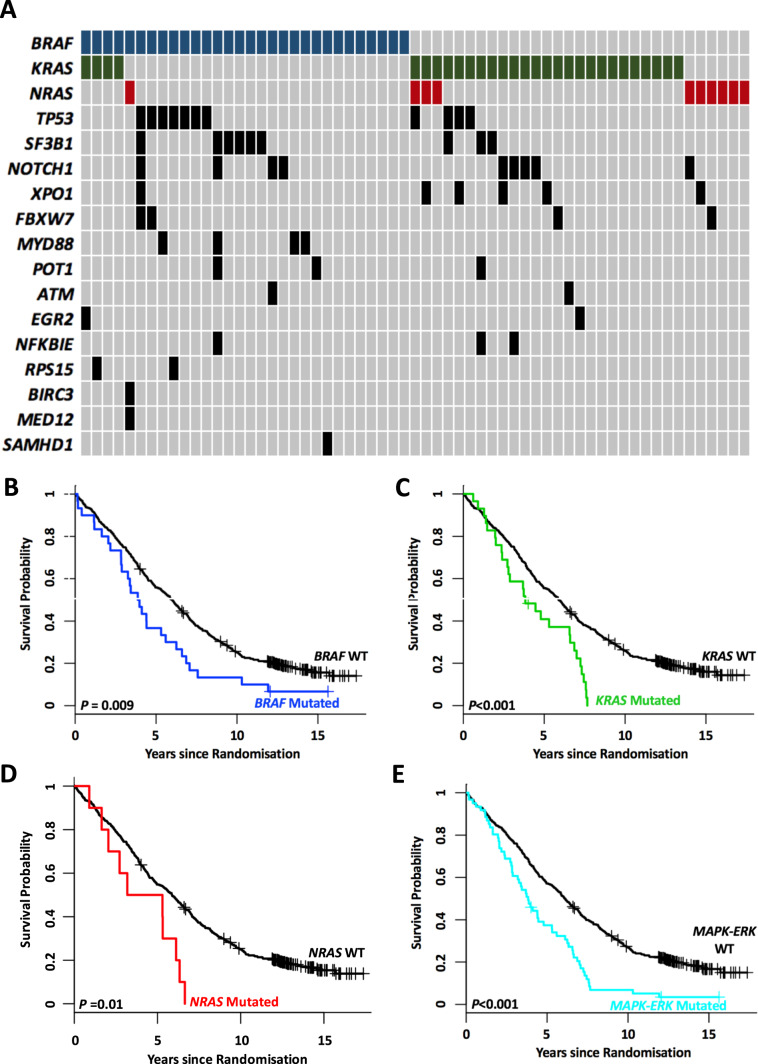
*MAPK-ERK* genes predict poor OS in CLL4. **a** Heat map of *BRAF* (blue)*, KRAS* (green)*, NRAS* (red) and co-mutated genes of *MAPK-ERK* mutated cases (black). Cases wild type for each gene represented by grey bars. Overall survival univariate KM plots for *BRAF* (**b**), *KRAS* (**c**), *NRAS* (**d**), and a combined variable of *APK-ERK* (**e**). Coloured line represents mutated cases, black line represents wild-type cases.

Mutations in *MAPK-ERK* genes, *BRAF* (38 mutations/30 cases), *KRAS* (34/29) and *NRAS* (11/10), were principally composed of specific hotspot variants (*BRAF*: p.G469A/E, *KRAS*: p.G13D, *NRAS*: p.Q61K/R) (Fig. S7), and the majority of *MAPK-ERK* mutated cases (87%) only harboured a mutation in one of these genes (Fig. 6a). Interestingly, *MAPK-ERK* mutated patients displayed an increased frequency of mutated genes and CNVs per case vs. *MAPK-ERK* wild-type patients (Fig. S20). In univariate analysis, each mutation was associated with a shorter median OS than wild type: *BRAF* (OS median: 3.92 years vs. 6 years, *P* *=* 0.009), *KRAS* (OS median: 3.83 years vs. 5.89 years, *P* < 0.001), and *NRAS* (OS median: 4.24 years vs. 5.88 years, *P* *=* 0.01) (Fig. 6b–d). Stratified <12% VAF vs. ≥ 12% VAF analysis indicated that the outcome of *KRAS* mutated cases was independent of VAF while shorter OS in *BRAF* mutated cases was associated with < 12% VAF (Fig. S16; Table S9). Taken together, *MAPK-ERK* mutations exhibited inferior OS compared with wild-type cases (OS median: 3.83 years vs. 6.10 years, *P* < 0.001), and were negatively associated with long-term survival (Odds ratio = 0.19, *P* *=* 0.0003) (Fig. 6e), with only 4/60 mutated cases defined as long-term survivors. Furthermore, *MAPK-ERK* mutated patients were more likely to carry IGHV-U genes (IGHV-U Odds Ratio = 4.29, *P* < 0.0001; IGHV homology >99% Odds ratio = 3.51, *P* *=* 0.0002), and significantly less likely to harbour del(13q) as a sole aberration (Odds ratio = 0.23, *P* < 0.0001, Table S10).

### Multivariate modelling identifies TP53ab, biallelic BIRC3, SF3B1, EGR2 and MAPK-ERK gene mutations as independent markers of inferior OS

Finally, we constructed comprehensive multivariate Cox Proportional Hazards models for PFS and OS (Table 2), which included those clinical and genetic variables significant in univariate analysis, as well as biallelic *ATM* and *BIRC3* as they emerged from our stratified 11q deletion analysis, and short telomeres based on our previous paper on the topic [32]. A backwards selection approach was applied, until all variables within the model had a *P* value < 0.05. For PFS, the final model was constructed from 225 patients and 210 events (274 were excluded due to missing data) and showed that *TP53*ab (HR = 4.98, *P* < 0.001), biallelic *BIRC3* (HR = 3.83, *P* *=* 0.004), short telomeres (HR = 1.96, *P* < 0.001), sole 11q deletion (HR = 1.82, *P* = 0.003), and increased prolymphocytes (HR = 1.51, *P* = 0.033) were independent markers of PFS. For OS, the final model was constructed from 391 patients and 323 events (108 observations were excluded due to missing data). *TP53*ab (HR = 4.25, *P* < 0.001), biallelic *BIRC3* (HR = 2.76, *P* *=* 0.004), mutations in *EGR2* (HR = 2.19, *P* *=* 0.015), *MAPK-ERK* genes (HR = 1.68, *P* = 0.002), *SF3B1* (HR = 1.54, *P* = 0.001), as well as IGHV-U genes (HR = 1.83, *P* < 0.001) and Binet stage B&C (HR = 1.45, *P* = 0.008), were all observed as independent markers of OS. This data extends on our univariate survival analysis, showing that cases with biallelic *BIRC3* deletions exhibit reduced PFS and OS, and that mutations in the *MAPK-ERK* pathway lead to reduced OS.

**Table 2:** Multivariate Cox model for overall survival and progression-free survival in CLL4.

Survival	Variable	HR	LCI	UCI	*P*
Overall	*TP53*ab	4.247	2.932	6.151	<0.0001
	Biallelic *BIRC3*	2.756	1.397	5.438	0.003
	*EGR2* mutated	2.188	1.167	4.099	0.015
	IGHV-U	1.831	1.417	2.364	<0.0001
	*MAPK-ERK* mutated	1.683	1.202	2.356	0.002
	*SF3B1* mutated	1.544	1.191	2.002	0.001
	Binet Stage B & C	1.454	1.102	1.918	0.008
	11q deletion	1.431	1.081	1.895	0.012
Progression-free	*TP53*ab	4.975	3.049	8.118	<0.001
	Short Telomeres	1.964	1.466	2.629	<0.001
	11q deletion	1.816	1.226	2.688	0.003
	Biallelic *BIRC3*	3.833	1.537	9.557	0.004
	Prolymphocytes	1.508	1.034	2.198	0.033

The OS model was built using the following starting variables: *MAPK-ERK*mut, *TP53*ab (after removal of < 12% *TP53* mutations), *EGR2*mut, *RPS15*mut, *NFKBIE*mut, *MYD88*mut, *SF3B1*mut, *NOTCH1* + 3′UTRmut, Binet Stage B&C, 11q deletion, biallelic *ATM*, biallelic *BIRC3*, sole 13q deletion, trisomy 12, IGHV-U. The final model for OS consisted of 391 patients and 323 events. The PFS model was built using the following starting variables: *TP53*ab, *EGR2*mut, biallelic *ATM*, biallelic *BIRC3*, 11q deletion without *ATM* or *BIRC3* mutations, sole 13q deletion, Short Telomeres, Prolymphocytes+ and IGHV-U. The final model for PFS consisted of 225 patients and 210 events. Variables for both OS and PFS MVA models were removed using the backwards selection method.

*HR* hazard ratio, *LCI* lower confidence interval, *UCI* upper confidence interval, *P* multivariate log rank *P* value.

## Discussion

We report targeted resequencing analysis of 22 genes known to be recurrently mutated in CLL in the UK CLL4 clinical trial. CLL4 represents an ideal candidate for such an analysis, with expansive clinical and biological description [8, 12, 13, 17, 21, 24, 26, 29, 31–33, 40, 41] and protracted clinical follow-up. Our study confirms previous studies incorporating samples from this patient cohort showing the impact of *TP53*ab on PFS and OS in MVA, *SF3B1, EGR2* [25, 26], *RPS15* [1, 24] and *NFKBIE* [25, 28, 42] mutations on OS in univariate analysis, with *SF3B1* and *EGR2* mutations retained as independent markers of OS in MVA.

The literature suggests that patients with *MAPK-ERK* mutations represent a biologically distinct subgroup, where *MAPK-ERK* mutations are frequently mutually exclusive, are enriched for trisomy 12, unmutated IGHV genes and other adverse biological markers (e.g. CD38, ZAP-70, CD49d), and are linked to inferior time to first treatment in retrospective cohorts [41, 43–45]. We now show the *MAPK-ERK* genes, *BRAF, KRAS* and *NRAS* (collectively representing 12.2% of patients) are also independently associated with short OS in a cohort of patients requiring treatment. Vendramini et al. showed a similar frequency of mutations in these genes (14%) [44], while Giménez et al. found that 5.5% of CLL cases harbours functionally deleterious mutations in 11 genes involved in the *MAPK-ERK* pathway [45], the latter likely reflects the early-stage composition of the cohort. In support of the biological impact of these mutated genes in CLL, (1) Analysis of mutated patients exhibit an enrichment of gene sets associated with transcriptional activation of the MAPK-ERK pathway [44], (2) preliminary in vitro analysis suggests cells from these patients are prone to killing with ERK inhibitors [45], (3) *BRAF* mutations accelerated disease progression in Eµ-TCL1 mice [46], (4) mutant *BRAF* has been implicated in venetoclax resistance [47], and (5) *KRAS* mutated cases associated with poor response to chemoimmunotherapy [27] and lenalidomide [48].

Screening for *TP53*ab using FISH and Sanger sequencing has known prognostic value [6, 8, 20, 31], and predicts for resistance to chemoimmunotherapy [49]. *TP53* mutations that present at low VAFs, below the detection limit of conventional Sanger sequencing may also be positively selected by chemotherapy, and also predict inferior survival, at least in retrospective, institutional cohorts [3, 5, 9]. The *TP53* Network of ERIC provide expansive guidelines on the most suitable approach for *TP53* mutational analysis, but also conclude that the clinical importance of low-level *TP53* clones remains an unresolved issue, requiring validation in clinical trials [49]. We demonstrated inferior PFS and OS only for those patients with ≥12% VAF *TP53* mutations, but we could not demonstrate inferior survival associated with cases harbouring <12% VAF *TP53* mutations, the inference perhaps is that these cases represent an intermediate-risk group. Given the unexpected nature of this finding, we also conducted stratified 17p deleted survival analysis, identifying the same result for <12% VAF *TP53* mutations without 17p deletion. Furthermore, we proceeded to show that our observation was not associated with any differences in the type of *TP53* mutation, their co-existence with other more clonal prognostically-important gene mutations or biological features, nor the enrichment of any specific treatment. As a consequence, we feel that our observation is technically sound, and warrants confirmation in further studies.

There remains disagreement regarding the relative clinical significance of deletion and mutation of the *BIRC3* and *ATM* genes, both mapping to the long arm of chromosome 11. The *ATM* gene is mutated in 30–40% of 11q deleted patients [11, 13], where it results in biallelic inactivation of *ATM*, driving an impaired DNA damage response [50]. The prognostic impact of *ATM* mutations is controversial in unselected cohorts [9], with the strongest impact when the wild-type allele is lost. In our study, whilst we triaged *ATM* mutations based on their putative pathogenicity, several are reported in both somatic (i.e. COSMIC) and germline (i.e. dbSNP, EXAC, ClinVar) databases, lending uncertainty to their prognostic impact. The sequencing of matched germ-line material would provide additional clarity, but was not possible due to the historical nature of CLL4. Preliminary studies support a pathogenetic role of *BIRC3* [16, 39], more recent studies provide less certainty. For example, in the RESONATE clinical trial [51] and the large retrospective study coordinated by ERIC [52], *BIRC3* mutations were not linked to inferior PFS or TTFT, respectively. Another comparator would be the RESONATE2 trial, which compared first line treatment with Ibrutinib vs. chlorambucil [53]. The 24 month PFS for 11q deleted patients in the Ibrutinib arm was 97%. Further studies are required to determine if the long-term outcome of biallelic *BIRC3* cases is equally good under modern small molecule inhibition. In our previous CLL4 analysis, we demonstrated that *BIRC3* dysfunction (defined as deletion AND/OR mutations of *BIRC3*) did not impact survival in 11q deleted CLL, while biallelic *ATM* lesions remained informative [12]. However, this analysis utilised Sanger sequencing, and hence only identified a small number of *BIRC3* mutations. Our current study, therefore aimed to expand the analysis with a larger patient cohort with significantly improved technology. This approach permitted the identification of a meaningful number of cases with loss and mutation of *BIRC3*. As neither *ATM* nor *BIRC3* mutations were linked to survival in univariate analysis, we performed a stratified analysis in 11q deleted cases. In so doing, we show that biallelic *BIRC3* cases have a further reduction in survival in comparison to sole 11q deleted cases and were found to be independent prognostic markers for PFS and OS in MVA. Finally, *ATM* and *BIRC3* mutated cases without 11q deletion have a similar survival to wild-type cases.

In conclusion, our study makes three main contributions to the field. We show an expansive analysis of the impact of clinico-biological disease features on the clinical importance of important gene mutations, including *SF3B1, EGR2* and the *MAPK-ERK* genes. Our analysis suggests that <12% VAF *TP53* mutations are an intermediate survival group. Finally, we show that biallelic *BIRC3* aberrations identify a novel patient subgroup with poor survival, inferior to those with 11q deletions alone. Taken together, we demonstrate that a more expansive genomic screening approach provides additional clinical information, thereby helping to establish the precise importance of genetic alterations in the context of other established and emerging biomarkers. Furthermore, our work will facilitate the development of international standards for the detection and interpretation of somatic mutations in CLL.

## Supplementary information

Supplementary Material

Supplemental Table S3

Supplemental Table S4

Supplemental Table S6
