# Supplementary material for: Clinical significance of TP53, BIRC3, ATM and MAPK-ERK genes in chronic lymphocytic leukaemia: data from the randomised UK LRF CLL4 trial
Source: Leukemia. 2020 Feb 3;34(7):1760–74. doi: 10.1038/s41375-020-0723-2 (PMC7326706; doi:10.1038/s41375-020-0723-2)
Supplement: Supplementary file 1 — Supplementary Material [file 41375_2020_723_MOESM1_ESM.docx]

# Supplementary Information

**Clinical Significance of *TP53, BIRC3, ATM* and *MAPK-ERK* genes in Chronic Lymphocytic Leukaemia: Data from the Randomised UK LRF CLL4 Trial**

Blakemore *et al.*

## Supplemental Figure Legends

**Figure S1. Overview of targeted re-sequencing strategy of the CLL4 cohort.**

Graphical abstract representing the key steps and analytical processes conducted for this targeted re-sequencing study of CLL4 cases. From top to bottom, 499 cases were sequenced for mutations in 22 key genes, followed by identification of 623 variants using strict bioinformatics cutoffs (see methods for more details). Orthogonal sequencing was conducted for 194 variants, with 100% of variants being validated, showing the effectiveness of the stringent bioinformatics approach. Assessment of the clonal architecture of mutations in CLL4 cases was carried, with key examples taken forward for survival analysis.

**Figure S2. Lymphocyte percentage vs. CD19 x CD5 positive CLL cell Bland-Altman analysis**

Comparison between Lymphocyte percentage and CD19 x CD5 flow cytometry data from CLL4 patients with both datasets available. Bland Altman plot comparing these differences, with the *y* axis representing the difference in percentage values, and the *x* axis representing the average of the two measurements.

**Figure S3. Mean Read Depth of the CLL4 TruSeq study.**

Scatterplot displaying the mean read depth ± standard deviation, achieved for each gene in the TruSeq Custom Amplicon Panel. Dotted line at 1000dp.

**Figure S4. Orthogonal sequencing validation of key variants in *TP53, SF3B1, NOTCH1,* and *ATM.***

Bland Altman plot showing the difference and average between the original TruSeq VAF and the orthogonal sequencing VAF.

**Figure S5 Distribution of variants across 22 genes in CLL4.**

Lolliplots representing the variants observed in the CLL4 TruSeq study. Green represents missense mutations, black truncating events and brown in-frame events. Lolliplots were created using the Mutation Mapper online tool.

**Figure S6. Multiple variants per gene in CLL4.**

Bar chart indicating the number of cases with ≧2 variants per gene.

**Figure S7. Distribution of BRAF mutations in CLL4.**

**A** Lolliplot depicting *BRAF* mutations observed in CLL4. Lolliplot created using the Mutation Mapper online tool. **B** Mutation frequency of each *BRAF* variant, ranked by from highest to lowest.

**Figure S8 Comparison between tumor associated VAFs and original PBMC VAFs in CLL4. A** Bland Altman plot of the mutations from 288 patients with tumor purity information available from CD19 CD5 flow cytometry data. **B** Bland Altman plot of 98 sub-clonal variants (<12% raw VAF) from patients with tumor purity information available from CD19 CD5 flow cytometry data.

**Figure S9 Progression-free Survival of mutated genes in CLL4.**

Kaplan Meier plots of 19 genes with sufficient mutated cases to assess survival impact. Red line represents mutated cases in each plot, with black lines representing wild type cases for that gene. Log rank *P* values included in the lower left inset of the Kaplan Meier graph for each gene.

**Figure S10 Overall Survival of mutated genes in CLL4.**

Kaplan Meier plots of 19 genes with sufficient mutated cases to assess survival impact. Red line represents mutated cases in each plot, with black lines representing wild type cases for that gene. Log rank *P* values included in the lower left inset of the Kaplan Meier graph for each gene.

**Figure S11 *MYD88* mutations in IGHV-M CLL do not associate with increased overall survival in CLL4.**

Kaplan Meier plot of *MYD88* mutated (red) vs. wild type (black) from IGHV-M CLL4 cases. Log rank *P* value included in the lower left inset of the Kaplan Meier graph.

**Figure S12 Co-occurrence of mutated genes with response in CLL4.**

Bar graph representing the change in mutation frequency between the NR/PD (green) and CR/NodPR (blue) response groups. Fisher’s Exact test with FDR testing used to compare the differences between the two groups. **Q*>*P* (*P*<0.05), § *P*<0.05.

**Figure S13 Co-occurrence of variables with disease transformation and long term survival in CLL4.**

**A** Bar graph representing the change in mutation frequency between all cases (green) and death from Richters Transformation (blue). **B** Bar graph representing the change in mutation frequency between the NR/PD (green) and CR/NodPR (blue) response groups. Fisher’s Exact test used to compare the differences between the two groups. **P*<0.05, ** *P*<0.01, *** *P*<0.001.

**Figure S14 In-going and out-going edges of *TP53* mutated cases in CLL4.**

The in-going and out-going edges of *TP53* mutated patient were drawn between *TP53* mutations and other mutated genes based on VAF. Coloured bars represent the various *TP53* mutated groups: Sole ≥12% VAF *TP53* (blue), ≥12% VAF *TP53* + del(17p) (red), sole <12% VAF *TP53* (green), <12% VAF *TP53* + del(17p) (purple). CLL4 identification numbers (including IGHV mutational status when available), were used to define the patients.

**Figure S15 Clinical relevance of <12% VAF *TP53* mutations in CLL4 stratified by FISH 17p deletion status**

**A** OS pairwise KM plot comparing ≥12% VAF *TP53* mutation with 17p deletion (red), sole ≥12% VAF *TP53* mutation (blue), sole <12% VAF *TP53* mutation (green), and *TP53*ab wild type patients (black). **B** PFS pairwise KM plot comparing ≥12% VAF *TP53* mutation with 17p deletion (red), sole ≥12% VAF *TP53* mutation (blue), sole <12% VAF *TP53* mutation (green), and *TP53*ab wild type patients (black). Inset table in A&B displays pairwise log rank *P* values between each variable vs. wild type.

**Figure S16 Impact of clone size on overall survival in CLL4.**

Pairwise Kaplan Meier plots for each gene with sufficient data in each group. >12% mutated cases (red), <12% mutated cases (green) were tested using pairwise Log rank *P* values vs. wild type cases for each gene (black) for *BIRC3* (**A**), *BRAF* (**B**), *KRAS* (**C**), *NOTCH1* (**D**), *POT1* (**E**), *SF3B1* (**F**). Pairwise *P* values vs. Wildtype presented as a table in the top right insert of the Kaplan Meier graph for each gene.

**Figure S17 Impact of clone size on progression-free survival in CLL4.**

Pairwise Kaplan Meier plots for each gene with sufficient data in each group. >12% mutated cases (red), <12% mutated cases (green) were tested using pairwise Log rank *P* values vs. wild type cases for each gene (black) for *BIRC3* (**A**), *BRAF* (**B**), *KRAS* (**C**), *NOTCH1* (**D**), *POT1* (**E**), *SF3B1* (**F**). Pairwise *P* values vs. Wildtype presented as a table in the top right insert of the Kaplan Meier graph for each gene.

**Figure S18 Shallow WGS showing breakpoint loci in biallelic *BIRC3* cases**

Chromosome 11 copy number alteration plots for *BIRC3*mut samples. X axis indicates chromosomal coordinate, Y-axis indicates the log2 ratio of read counts per 5kb bin, where blue points are individual genomic bins and orange bars indicate bins pooled into segments with the same copy number. Orange bars below log2 ratio of 0 represent deletions, those that are significant are highlighted in red. A-D) samples 4104, 4145, 4566 and 4769, respectively.

**Figure S19 Combined pairwise Kaplan Meier plots: Importance of biallelic loss of *ATM* and *BIRC3* in CLL4.**

**A** OS pairwise KM plot comparing mutated *ATM* and *BIRC3* in the context of 11q deletion. **B** PFS pairwise KM plot comparing mutated *ATM* and *BIRC3* in the context of 11q deletion. Inset table in A&B displays pairwise log rank *P* values between each variable vs. wild type.

**Figure S20 *MAPK-ERK* mutated cases show increased number of mutated genes and CNVs vs. *MAPK-ERK* wild-type cases.**

Bar graph showing the mutated genes/CNVs *MAPK-ERK*mut vs. *MAPK-ERK*wt cases. *TP53*ab cases excluded from this analysis. Student’s T-test conducted between the two groups.

## Supplemental Tables & Figures

**Table S1. Comparison of the characteristics of the patient cohort profiled, to the full LRF CLL4 trial cohort.**

**
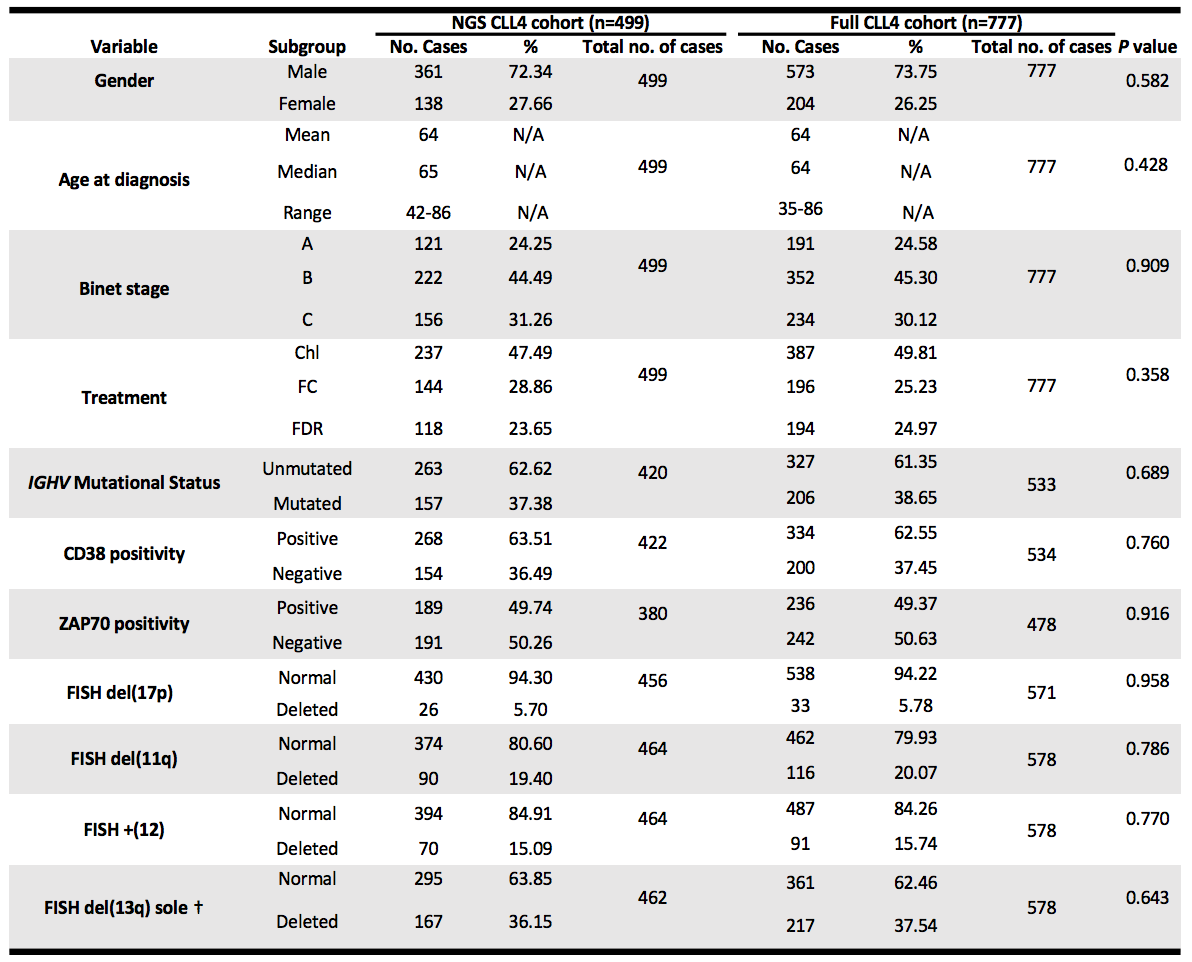
**

*Age and disease stage were both assessed at trial entry. ZAP70 and CD38 expression was determined as previously described (1, 2) where 10% and 30% positive cells were classed as positive, respectively. †The presence of a 13q deletion as a sole abnormality using a standard FISH panel. *P* values were calculated from 2 x 2 or 2 x 3 Chi-squared tests (Fisher exact test was used when observations were < 5). (significance level = 0.05).

**1.** Best OG, Ibbotson RE, Parker AE, Davis ZA, Orchard JA, Oscier DG. ZAP-70 by flow cytometry: a comparison of different antibodies, anticoagulants, and methods of analysis. Cytometry B Clin Cytom 2006; 70(4): 235-241.

**2.** Oscier DG, Gardiner AC, Mould SJ, Glide S, Davis ZA, Ibbotson RE, et al. Multivariate analysis of prognostic factors in CLL: clinical stage, IGVH gene mutational status, and loss or mutation of the p53 gene are independent prognostic factors. Blood 2002; 100(4): 1177-1184.

**Table S2. The CLL4 TruSeq Custom Amplicon panel design.**


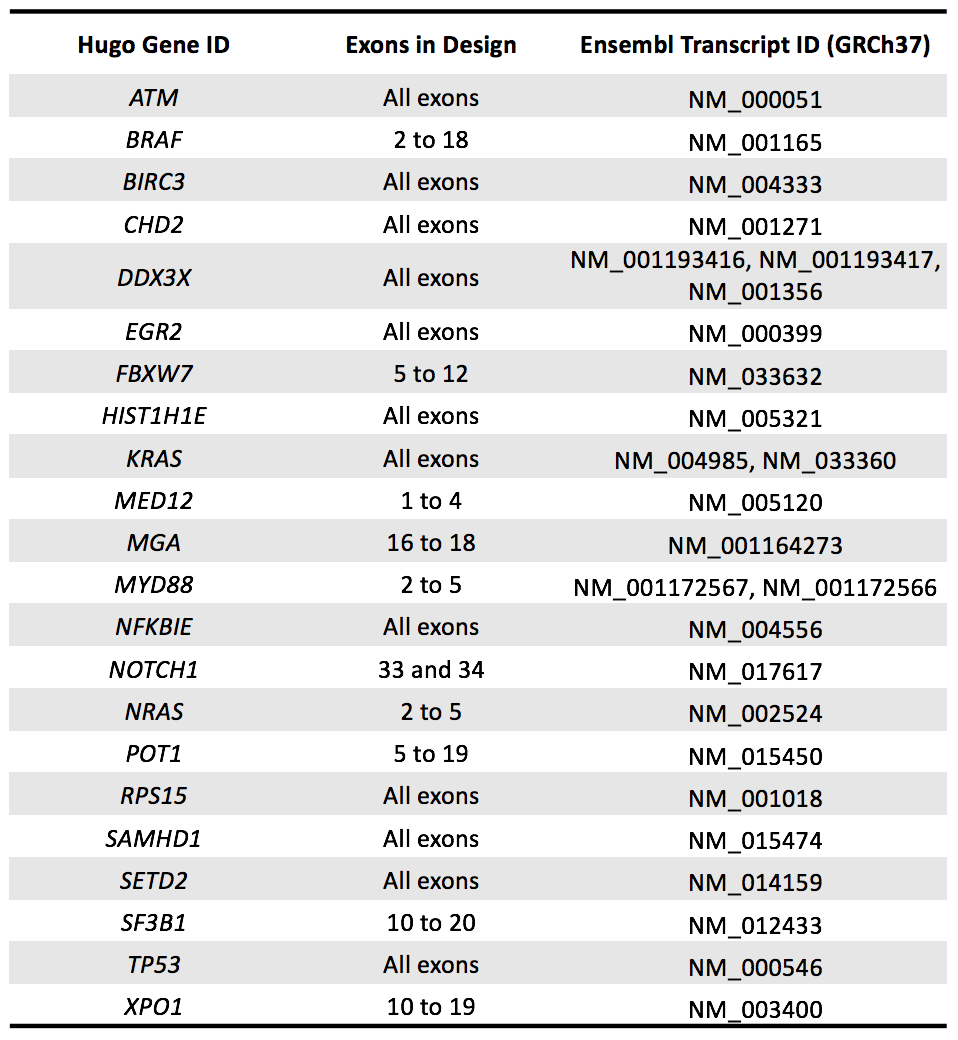


**Table S5. Recurrent mutated gene power calculation using TumorPortal**


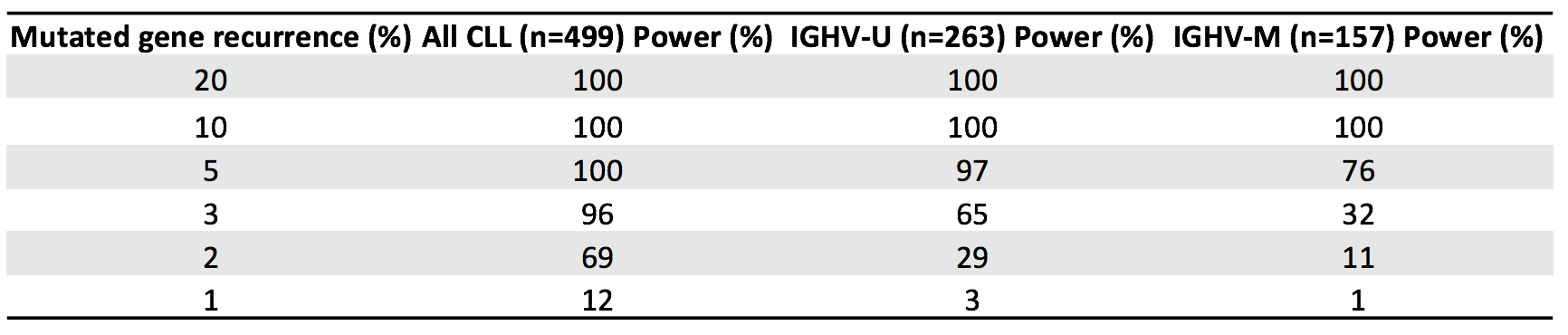


**Table S7. <12% *TP53* Co-occurrence analysis with all mutated genes, CNAs, clinical and biological features in CLL4.**

**Table S8. In-going/Out-going edges of *TP53* mutated cases
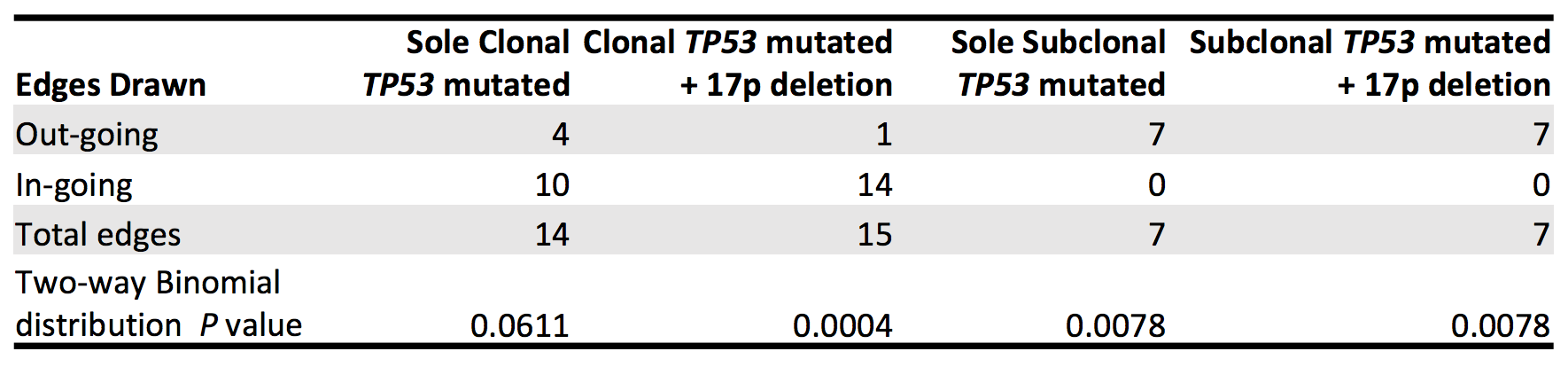
**

**Table S9. <12% *BRAF* Co-occurrence analysis with all mutated genes, CNAs, clinical and biological features in CLL4.**

**Table S10. MAPK-ERK Co-occurrence analysis with all mutated genes, CNAs, clinical and biological features in CLL4.**

**Figure S1.**

**Figure S2.**

**
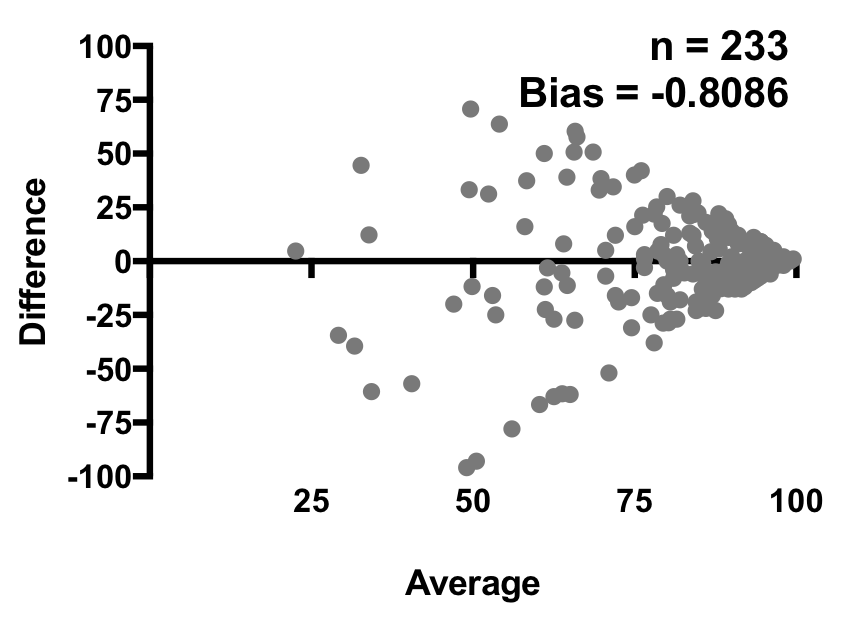
**

**Figure S3.**

**Figure S4.**

**Figure S5**

**Figure S6.**

**Figure S7.**

**Figure S8.**

**Figure S9.**

**Figure S10.**

**Figure S11.**

**Figure S12.**

**Figure S13.**

**Figure S14.**

**Figure S15.**

**Figure S16.**

**Figure S17.**

**Figure S18.**

**Figure S19.**

**Figure S20**
